# Uncoupling Protein 2 and 4 Expression Pattern during Stem Cell Differentiation Provides New Insight into Their Putative Function

**DOI:** 10.1371/journal.pone.0088474

**Published:** 2014-02-11

**Authors:** Anne Rupprecht, Dana Sittner, Alina Smorodchenko, Karolina E. Hilse, Justus Goyn, Rudolf Moldzio, Andrea E. M. Seiler, Anja U. Bräuer, Elena E. Pohl

**Affiliations:** 1 Institute of Physiology, Pathophysiology and Biophysics, University of Veterinary Medicine, Vienna, Austria; 2 Institute of Cell Biology and Neurobiology, Charité – Universitätsmedizin, Berlin, Germany; 3 Department of Experimental Toxicology and ZEBET, German Federal Institute for Risk Assessment (BfR), Berlin, Germany; 4 Institute of Medical Biochemistry, University of Veterinary Medicine, Vienna, Austria; Goethe University, Germany

## Abstract

Apart from the first family member, uncoupling protein 1 (UCP1), the functions of other UCPs (UCP2-UCP5) are still unknown. In analyzing our own results and those previously published by others, we have assumed that UCP's cellular expression pattern coincides with a specific cell metabolism and changes if the latter is altered. To verify this hypothesis, we analyzed the expression of UCP1-5 in mouse embryonic stem cells before and after their differentiation to neurons. We have shown that only UCP2 is present in undifferentiated stem cells and it disappears simultaneously with the initiation of neuronal differentiation. In contrast, UCP4 is simultaneously up-regulated together with typical neuronal marker proteins TUJ-1 and NeuN during mESC differentiation in vitro as well as during murine brain development *in vivo*. Notably, several tested cell lines express UCP2, but not UCP4. In line with this finding, neuroblastoma cells that display metabolic features of tumor cells express UCP2, but not UCP4. UCP2's occurrence in cancer, immunological and stem cells indicates that UCP2 is present in cells with highly proliferative potential, which have a glycolytic type of metabolism as a common feature, whereas UCP4 is strongly associated with non-proliferative highly differentiated neuronal cells.

## Introduction

The subfamily of uncoupling proteins (UCP1-UCP5) belongs to the superfamily of mitochondrial carriers that are alleged to shuttle metabolic substrates across the mitochondrial inner membrane [1]. In contrast to other members of the family, UCPs were shown to mediate a proton leak across the membrane, which was demonstrated for all UCPs via different experimental systems ([2]–[8]). In the case of UCP1, proton transport in the presence of long-chain fatty acids (FA) was linked to non-shivering thermogenesis [9], [10]. The exact function(s) for UCP2-UCP5 remains elusive although several interesting hypotheses exist. The most widespread one is that UCPs regulate reactive oxygen species (ROS) production in mitochondria. However, the fact that different UCPs (for example “brain”-associated UCP2, UCP4 and UCP5) with the same putative proton transport function are present in the same tissue has until now remained insufficiently explained or confirmed.

In 1997, UCP2 was discovered and its ubiquitous expression was mainly postulated on the basis of mRNA distribution [11], [12]. However, mRNA expression alone obviously does not mirror the protein distribution correctly due to its posttranslational regulation and the protein's short life time [13], [14]. These factors, together with unverified antibodies for UCP2 and other uncoupling proteins, seem to cause persistent confusion about UCP distribution in tissues, hindering the investigation of their specific function. We recently described the high abundance of UCP2 in cells with high proliferative and synthetic activity such as activated T-lymphocytes and haematopoetic stem cells [12], [15]. Using Western Blot analysis we have also demonstrated that only UCP4 and not UCP2 is reliably present in mice neurons [16], [17]. These data support the findings of some other research groups that have described the presence of UCP2 at the protein level in immune and pluripotent stem cells [13], [18]–[20] as well as in cancer cells [21]–[23]. However, the investigation of UCP2 functions in the brain continues to be an issue of scientific discussion [24]–[27].

Since knowledge about the correct protein localization seems to be a crucial prerequisite for the recognition of UCP function(s), we have now applied a dynamic approach which allows us to follow the protein expression under changing developmental and metabolic conditions. We initiated the differentiation of murine embryonic stem cells into neurons and analyzed the expression of UCP subfamily members. Our results strengthen the hypothesis that the expression of UCP2 is tightly connected to the cell metabolic state and thereby changes simultaneously upon its alteration.

## Methods

### Murine embryonic stem cells

Undifferentiated murine embryonic stem cells (mESCs, clone D3, [28] were routinely cultured in Dulbecco's modified Eagle's medium (DMEM) with 4500 mg/l D-glucose and without sodium pyruvate (Invitrogen, Karlsruhe, Germany) containing 15% fetal calf serum (HyClone™ / Thermo Scientific™, Bonn, Germany), 2 mM glutamine (Invitrogen), 1% non-essential amino acids (Invitrogen), antibiotics (50 U/ml penicillin, 50 µg/ml streptomycin; Invitrogen) and 0,1 mM β-mercaptoethanol (Sigma-Aldrich, Taufkirchen, Germany) as described previously [29]. Cells were passaged every 2–3 days and maintained in a humidified atmosphere at 37°C and 5% CO_2_. To prevent differentiation, 1000 U/ml murine leukemia inhibitory factor (Millipore, Schwalbach, Germany) were added to the medium.

Initiation of neural differentiation and maintenance of differentiated cells were performed as previously described with minor modifications [30]. In brief, cells from a high-density culture were seeded on poly-L-ornithine (15 µg/ml, Sigma-Aldrich) coated dishes at a density of 0.5×10^4^ cells/cm^2^ in N2B27 medium (50% DMEM/F12 with sodium pyruvate and GlutaMax (Invitrogen) supplemented with 1×N2 supplements (Invitrogen), 300 µg/ml bovine serum albumin fraction V (Sigma-Aldrich), 20 µg/ml insulin (Sigma-Aldrich), antibiotics (100 U/ml penicillin, 100 µg/ml streptomycin; Invitrogen), and 50% Neurobasal® medium (Invitrogen) with 1xB-27® (Invitrogen) and 0.1 mM β-mercaptoethanol (Sigma-Aldrich)) supplemented with 1 µg/ml laminin (Sigma) and 0.2% fetal calf serum (Invitrogen). Cells were maintained in a humidified atmosphere at 37°C and 5% CO_2._ On day 5 and 7 of differentiation, the medium was replaced with N2B27 medium supplemented with 1 µg/ml laminin. Starting at day 9, N2B27 medium was supplemented with 10 ng/ml human basic fibroblast growth factor (Strathmann Biotec AG, Hamburg, Germany) instead of laminin and changed every 2–3 days.

### Mouse neuroblastoma (N18TG2) and mouse microglia (BV-2) cell lines

N18TG2 cells (Deutsche Sammlung von Mikroorganismen & Zellkultur GmbH (DSMZ), Braunschweig, Germany) and BV-2 cells (Banca Biologica e cell Factory, Genova, Italy) were kept in 25 cm^2^ cell culture flasks with 5 ml medium in an incubator at 37°C, 5% CO_2_ and 100% humidity and split when reaching confluence. Cell culture media contained DMEM (4.5 mg/ml glucose) supplemented with either 9.7% fetal bovine serum, 3.85 mM glutamine and 1.94 mM sodium pyruvate (N18TG2 cells) or 9.8% fetal bovine serum and 3.92 mM glutamine (BV-2 cells) (all obtained from Sigma-Aldrich). Prior to experiments, cells were collected by centrifugation for 10 min at 178 g and re-suspended in serum-free media with afore mentioned concentrations of glutamine and sodium pyruvate. In addition, 2% B27® without antioxidants (Invitrogen) was added to the medium. Cells were cultivated in 6 well plates with 2 ml medium per well for another 48 h before the experiments.

### Animals and tissue samples

This study was carried out in strict accordance with the recommendations specified in the European guidelines (2010/63/EU) for the use of laboratory animals. The protocol was approved by the Committee on the Ethics of Animal Experiments (Landesamt für Gesundheit und Soziales, Berlin (LAGeSo); permit number: T0108/11). Pregnant, postnatal and adult C57BL/6 mice obtained from the central animal facility at Charité – Universitätsmedizin's Research Institutes for Experimental Medicine, were kept under standard laboratory conditions (12 hour light/dark cycle; (55±15)% humidity; (24±2)°C room temperature and water ad libitum, enriched and grouped). Pregnant and postnatal animals were sacrificed by decapitation; all efforts were made to minimize suffering. For one sample, six whole embryos (E8–E9), embryonic heads (E10–E12) or isolated organs from embryonic/young mice were collected. Samples were frozen in liquid nitrogen and stored at −80°C until protein or RNA isolation.

### Quantitative real time PCR (qRT PCR)

Total RNA from murine tissue samples and cultivated cells was extracted using TRIzol® reagent (Invitrogen). Production of cDNA was completed using the “High Capacity cDNA reverse Transcription kit” (Applied Biosystems, Foster City, CA, USA). Reverse transcriptase qRT PCR was performed with the following gene expression assays (Applied Biosystems): Mm00627598_m1, Mm01277266_m1, Mm00488302_m1, 4352932E and ID 4352933E for UCP2, UCP4, UCP5, GAPDH and β-actin respectively. For HPRT, separate primer and probe were used (Primer Mix: for 5′-ATCATTATGCCGAGGATTTGGAA-3′; rev 5′-TTGAGCACACAGAGGGCCA-3′ and probe 5′-TGGACAGGACTGAAAGACTTGCTCGAGATG-3′). The PCR was run on the ABI PRISM™ 7700 Sequence Detection System (Applied Biosystems) and the data obtained were processed by ABI PRISM software. Standard curves were produced with serial dilutions of cDNA from mouse neocortex with amplification efficiency between 90 and 100%. Data were normalized to two different housekeeping genes (GAPDH and HTPR), which produced similar results. Each result is represented by mean and SD from at least three separate experiments.

### Western blot analysis

The collection of total cellular protein from tissue and cell culture samples and Western blot (WB) analysis for UCP4 and UCP2 was performed as described previously [12], [16]. For all WB analyses 20 µg total protein per lane from the cell or tissue sample were loaded on the gel. To verify protein loading and for additional information, membranes were stripped, re-blocked, incubated with diluted antibody in blocking solution, incubated with a secondary antibody as described in detail in Smorodchenko et al. 2009 [16]. The following antibodies were used (dilution in brackets): voltage dependent anion channel, VDAC (Abcam, Cambridge, UK; ab14734; 1∶5000), GAPDH (Sigma-Aldrich; G8795, 1∶10000), succinate dehydrogenase subunit A, SDHA (Abcam; ab14715; 1∶10000), heat shock protein 60, Hsp60 (Abcam; ab59457; 1∶10000), pluripotency marker octamer binding transcription factor 3/4, Oct 3/4 (Santa Cruz Biotechnology, CA, USA, sc-9081; 1∶1000), neuronal marker TUJ-1 (Covance, Emeryville, CA, USA; MMS-435P; 1∶2500), 68 kDa neurofilament, NF (Abcam; ab7255; 1∶5000), β-actin (Sigma-Aldrich; A5441; 1∶5000), UCP1 (Sigma-Aldrich; U6382; 1∶1000). Affinity-purified polyclonal antibodies directed against mUCP2, mUCP3, mUCP4 and mUCP5 originated from our own laboratory [12], [16] and were used in dilution 1∶1000 if not otherwise indicated. Figure S1 shows the specificity of the antibody directed against mUCP3.

### Immunocytochemistry (ICC)

Cells plated on coverslips were fixed in ice cold 4% PFA for 25 min, washed three times in 0.1 M PBS and incubated with blocking solution containing 10% fetal calf serum (Biochrom, Berlin, Germany) and 0.05% Triton X-100 (Sigma-Aldrich) for 1 h at RT. Thereafter, cells were labeled overnight at 4°C using antibodies described above. The dilutions of antibodies for immunocytochemistry were: 1∶400-1∶1000 for anti-UCP4 and 1∶1000 for MAP2 (Sigma-Aldrich). After washing the cells with PBS, samples were incubated for 1 h at RT with the appropriate goat anti-mouse IgG Alexa-488 and goat anti-rabbit IgG Alexa-568 (Invitrogen) secondary antibodies, diluted 1∶1000 in blocking solution for 1 h at RT. After rinsing in PBS, coverslips were embedded in mounting medium containing DAPI (Vectashield; Vector Laboratories, Burlingame, CA, USA), dried and stored at 4°C.

### Immunohistochemistry (IHC)

Naïve C57BL/6 mouse was deeply anaesthetized with a mixture containing ketamine (Pfizer, Karlsruhe, Germany) and xylazine (Rompun@, Bayer, HealthCare, Leverkusen, Germany), then transcardially perfused with 20 ml ice-cold 0.1 M PBS and 20 ml 4% PFA. Brains were removed and fixed in 4% PFA. For confocal microscopy, the organs were washed in PBS for 1 hour. 50 µm coronal sections were prepared using vibrating blade microtome Leica VT1000 S (Germany). The sections were first permeabilized and blocked for non-specific binding using 0,5% Triton X-100 and 10% normal donkey serum (Vector Laboratories). Then the sections were incubated overnight with the primary antibodies against UCP4 (1∶1000); neuron nuclear protein, NeuN (1∶500; Millipore, Schwalbach, Germany), and neuronal migration protein doublecortin, Dcx (1∶100; Santa Cruz Biotechnology). Negative control sections were incubated in a similar manner without a primary antibody. Secondary antibodies donkey anti-rabbit IgG Alexa-488, donkey anti-mouse/rat IgG Alexa-633 and donkey anti-goat Alexa 594; (dilution 1∶1000, Invitrogen) in blocking solution were applied for 90 minutes at RT. After rinsing in PBS, tissue sections were coverslipped with Immu-Mount (Thermo Scientific, Waltham, MA, USA).

For light microscopy, brain sections were prepared in the same manner. Then the sections were incubated with 3% H_2_O_2_ for blocking of endogenous peroxidase, washed three times with 0.1 M PBS, and soaked for 1 hour in 10% normal goat or donkey serum to block non-specific binding. Thereafter, the free floating sections were incubated overnight at 4°C with primary antibodies diluted as described above in 1% serum and 0.5% Triton X-100. As secondary antibodies, biotinylated anti-rabbit IgG, anti-mouse IgG and anti-goat IgG (Vector Laboratories, Burlingame, CA, USA) were incubated in dilution 1∶1000 for 2 h at RT. Next, sections were pre-incubated with ABC-Elite (Vector Laboratories) and developed with 0.03% H_2_O_2_ and 1% 3,3′-diaminobenzidine tetrahydrochloride (DAB; Sigma-Aldrich). After rinsing in PBS, tissues were mounted on slides, dehydrated through a graded series of ethanol, cleared in xylene, and coverslipped with Entellan® (Merck, Darmstadt, Germany).

### Light- and confocal microscopy

Confocal microscopy was performed using an inverse confocal microscope (TCS SP5 Leica Microsystems) equipped with argon and helium-neon lasers with excitation wavelength 488 nm, 543 nm and 633, respectively. Image processing was performed with Leica Confocal Software and Image J. Light microscopy was performed as described in Smorodchenko et al., 2009 [16].

## Results

### UCP2 is expressed in mouse embryonic stem cells (mESCs)

**Figure 1:**
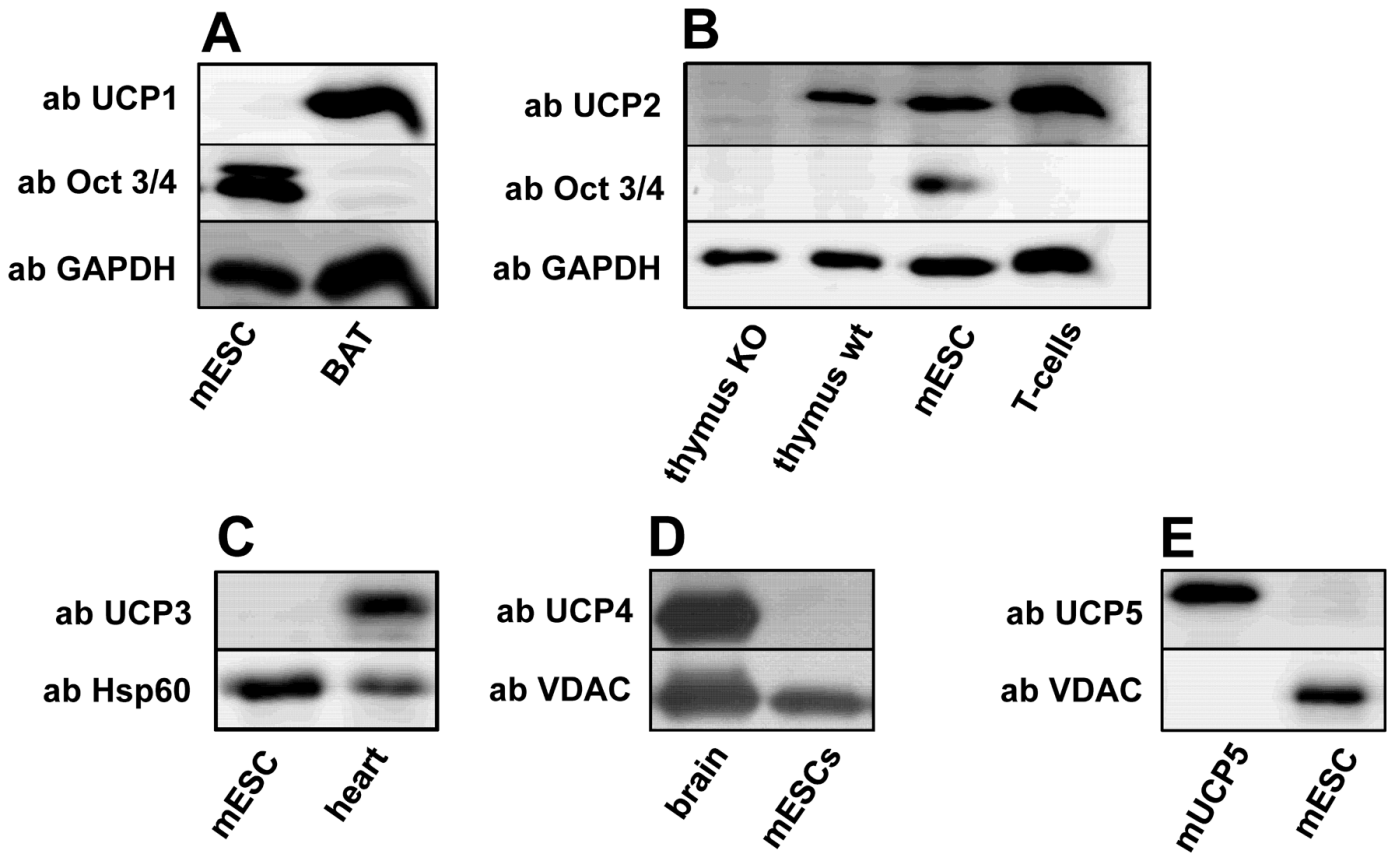
Embryonic stem cells express the pluripotency marker Oct 3/4 and uncoupling protein 2 simultaneously. A–E. Representative Western blot images showing UCP expression in mESCs using antibodies against UCP1 (A), UCP2 (B), UCP3 (C), UCP4 (D) and UCP5 (E). Brown adipose tissue (BAT), activated T-cells, brain from adult mice and recombinant mUCP5 were used as positive controls for the respective protein antibodies. Gels were loaded with 20 µg protein per lane. Antibodies directed against VDAC, GAPDH and Hsp 60 were used to visualize the respective proteins as loading controls. mESCs from at least 3 different passages were analyzed in each experiment.

Previously, we and other research groups have reported that UCP2 is expressed in haematopoietic and human pluripotent stem cells [15], [19], [20]. To confirm the common expression of UCP2 in stem cells, we now tested the protein abundance in mESCs. Using the pluripotency marker Oct 3/4 we first verified that mESCs were undifferentiated (Fig. 1A and 1B). The total cell protein extract from mESCs was analyzed by Western blot for the presence of UCP1-UCP5. Figure 1B shows a representative WB confirming the presence of UCP2 in mESCs. Neither UCP1 (Fig. 1A), UCP3 (Fig. 1C), UCP4 (Fig. 1D) nor UCP5 (Fig. 1E) were detected. As positive controls brown adipose tissue (BAT, for UCP1), activated T-cells (for UCP2, [12]), heart (for UCP3) and brain tissues (for UCP4, [16]) from mouse as well mUCP5 recombinant protein [16] were used.

### Analysis of UCP mRNA expression at different stages of differentiation

**Figure 2:**
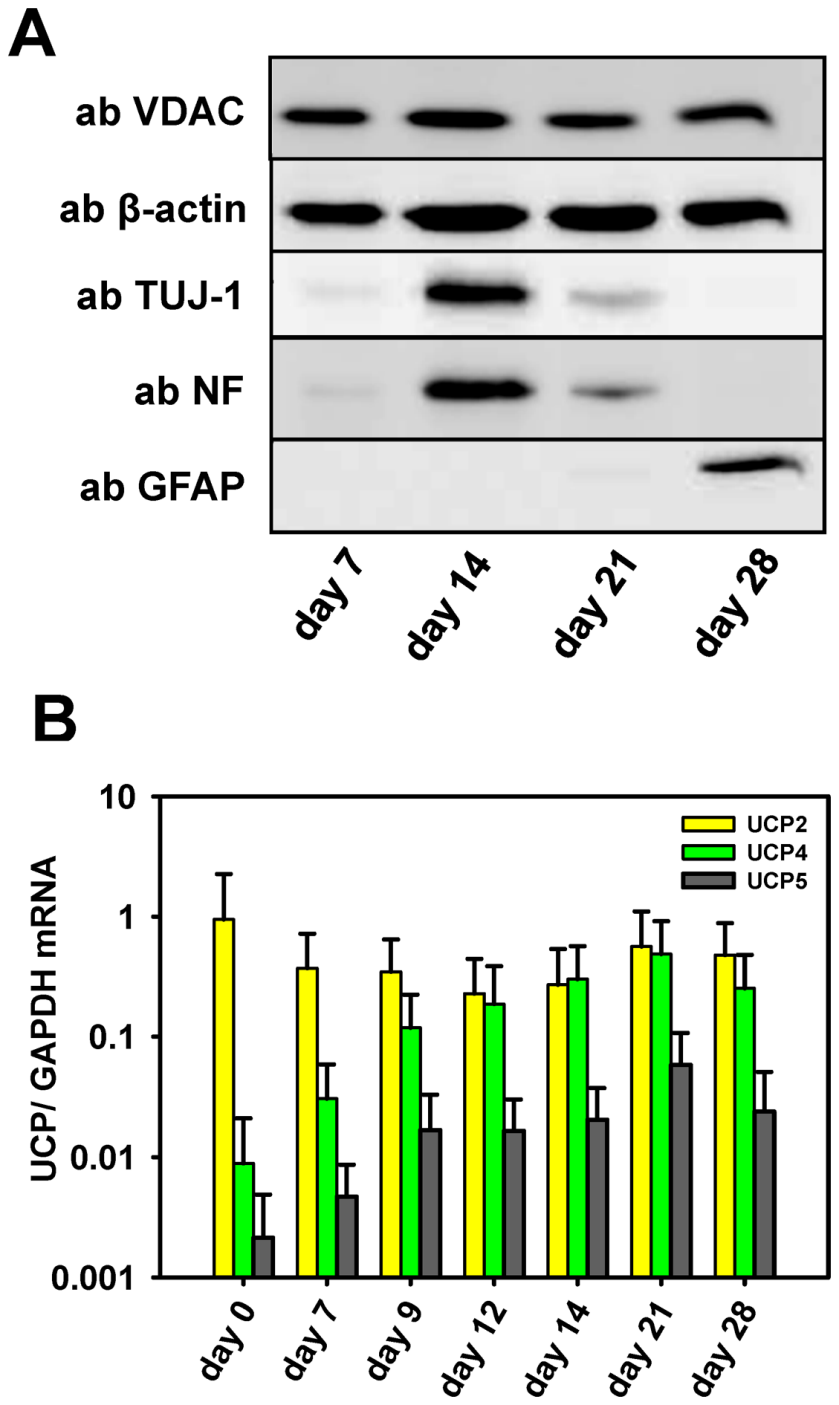
Expression of neuronal/glial markers and UCP mRNA during neuronal differentiation. A. Representative Western blots show the time-dependent expression of neuronal (TUJ-1 for young and NF for adult neurons) and astrocyte (GFAP) markers during mESCs differentiation in culture. Gels were loaded with 20 µg protein per lane. B. Real-time PCR analysis of mESCs shows the amount of UCP2, UCP4 and UCP5 mRNA relative to mRNA amounts of the housekeeping gene GAPDH at different time points during neuronal differentiation. Each data point represents the mean value and SD of 3 independent differentiation experiments.

**Figure 3:**
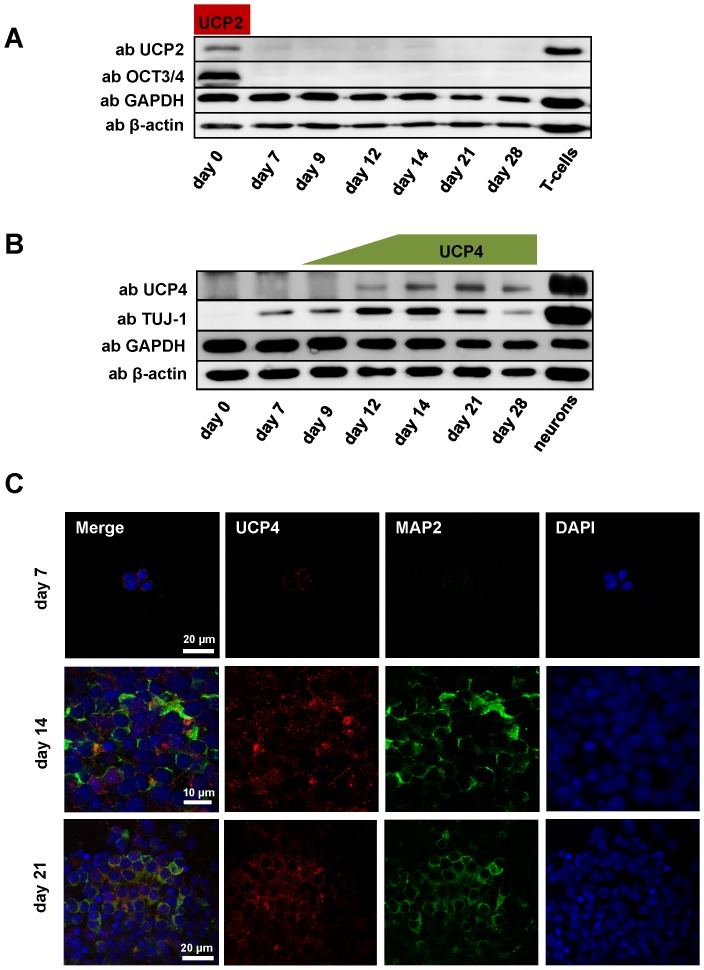
Neuronal differentiation coincides with a suppression of UCP2 and an induction of UCP4 expression. A–B. Representative Western blots of UCP2 (A) and UCP4 (B) expression during the differentiation of mESCs in culture. Activated T-cells and primary neuronal cultures (13 days) were used as positive controls. Gels were loaded with 20 µg protein per lane. Cells were collected at different time points from at least three independent differentiation experiments. C. Representative fluorescent images showing the time course of UCP4 and MAP2 expression during differentiation of mESCs to neurons. Primary antibodies were visualized by Alexa-488 (MAP2, green) and Alexa-567 (UCP4, red) respectively. Cell nuclei were counterstained with DAPI (blue).

Because both UCP2 and UCP4 were reported to be abundant in neurons, we analyzed their expression at mRNA and protein levels in mESC cultures at day 0 (cells before the initiation of differentiation) and day 7, 9, 12, 14, 21 and 28 of neuronal differentiation (Fig. 2 and 3). mESCs were differentiated into neurons according to the protocol described in Visan et al., 2012 [30]. Consistent with this differentiation method MAP2-positive neurons dominated in the cell culture between days 12 and 16 [30] and were confirmed therein by WB analysis using the neuron-specific markers TUJ-1 and NF (Fig. 2A). The up-regulation of GFAP at day 28 showed the expected increase of astrocytes in the cell culture [30].

Fig. 2B represents the quantitative mRNA measurements of UCP2, 4, and 5 in relation to the expression of the housekeeping gene GAPDH. The data show that UCP2 mRNA levels in cells before the initiation of differentiation were comparable to the mRNA levels of GAPDH and 100-fold higher than that of UCP4 (Fig. 2B). Furthermore, whereas the high UCP2 mRNA levels remained nearly constant over the whole period of differentiation, UCP4 mRNA levels steadily increased. The increase was already evident at day 7 and reached an approximately 100-fold higher level at day 21 after initiation of neuronal differentiation (Fig. 2B, grey bars). The highest levels of UCP4 mRNA observed, coincided with the dominance of neurons in culture (Fig. 2A). UCP5 mRNA expression during differentiation changed similarly to the UCP4 expression, however, the absolute mRNA levels were essentially lower. This may be the main reason why we could not detect UCP5 at protein level in WB.

### Differentiation of mESCs to neurons results in disappearance of UCP2 and an increase in UCP4 expression

In contrast to its nearly constant mRNA levels, UCP2 promptly disappeared on the protein level with the initiation of neuronal differentiation (Fig. 3A). UCP4 protein expression increased until day 14 and stayed at a comparably high level afterwards in agreement with its mRNA expression (Fig. 3B). The high abundance of UCP4 at day 14 coincided with the up-regulation of the neuronal marker TUJ-1, which reached its highest expression level around day 14 of differentiation (Fig. 2A, 3B). The comparison of mRNA and protein levels clearly shows that only the UCP4 mRNA values above the ratio UCP4 mRNA/GAPDH mRNA  = 0.01 ensure the presence of UCP4 at protein level. In contrast, generally higher UCP2 mRNA levels are not a prerequisite for protein detection. This supports the strong posttranslational regulation of UCP2 (but not of UCP4!) as previously described [13].

Immunocytochemical staining of cells at days 7, 14 and 21 of neuronal differentiation showed the results which confirm our WB data. UCP4 and the neuronal marker MAP2 are only slightly expressed at day 7 (Fig. 3C). The maximum of the expression of both markers was measured around day 14 which coincides with data obtained in embryonic tissue [16]. The decrease in fluorescence intensity of both markers toward day 21 can be explained by the increase of astrocytes in cell culture [30].

### UCP4 expression starts simultaneously with neuronal marker expression during murine neuronal development

**Figure 4:**
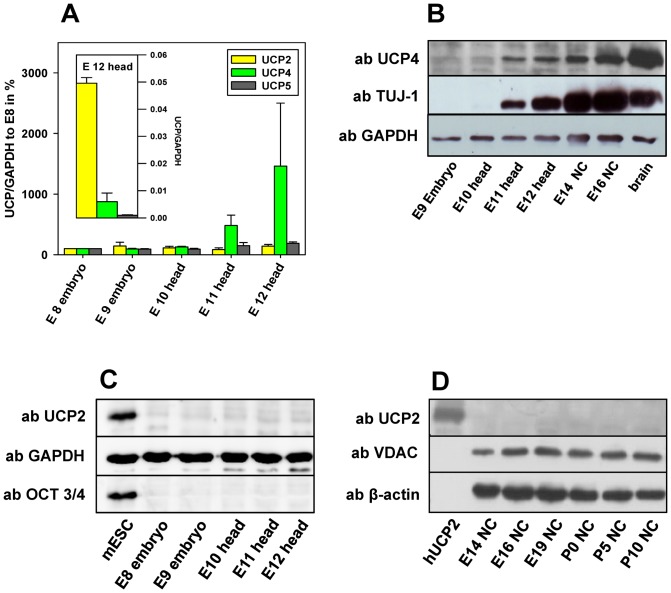
UCP4 expression starts simultaneously with the expression of neuronal markers. A. UCP2, UCP4 and UCP5 mRNA levels during neuronal development analyzed by quantitative PCR. UCP mRNA levels in mouse head are shown as a ratio to GAPDH at embryonic day 12 (E12; inset) and as a ratio (UCP mRNA)/(GAPDH mRNA) at different days to (UCP mRNA)/(GAPDH mRNA) at E8. B. Representative Western blot indicates the simultaneous start of UCP4 protein expression with the expression of the neuronal marker TUJ-1. C-D. Representative Western blots demonstrate that UCP2 is not present at the protein level in the tested embryonic tissue (C) as well as in young postnatal neocortical brain tissue (NC) (D). Gels were loaded with 20 µg protein per lane. GAPDH, β-actin and VDAC were used as loading controls. At least 3 samples of pooled embryonic and postnatal tissue from at least 6 mice were analyzed at each condition (Experiments A–D).

To support the data revealed in an *in vitro* model of neuronal differentiation, we analyzed the expression of UCP2, UCP4 and UCP5 during neuronal development in mouse embryos at different gestation stages (Fig. 4). Quantitative mRNA analysis showed that the absolute UCP2 mRNA levels were always higher than the corresponding UCP4 mRNA levels at all analyzed embryonic stages (Fig. 4A, inset, shown here for day 12). The relative levels of UCP2 and UCP4 mRNA were determined in relation to the expression of the housekeeping gene GAPDH and were then compared to the amount of mRNA at embryonic day 8 (E8). Whereas UCP2 mRNA level remained unchanged from E8 to E12, UCP4 mRNA showed a noticeable increase starting from E11 (Fig 4A). In accordance with the mRNA data, UCP4 protein expression started simultaneously with the neuronal marker TUJ-1 at E11 (Fig. 4B). In contrast, UCP2 could neither be detected at the embryonic stage (Fig. 4C) nor in early postnatal neocortical tissue (NC, Fig. 4D). Relative UCP5 mRNA expression shows only a slight increase at E11 and E12 (Fig. 4A). However, the UCP5 mRNA levels compared to GAPDH mRNA levels were below 0.001. This may explain why it could not be detected at protein level by WB.

### UCP4 is not expressed in Dcx^+^/NeuN^−^ neuroblasts in the adult subventricular zone (SVZ)

**Figure 5:**
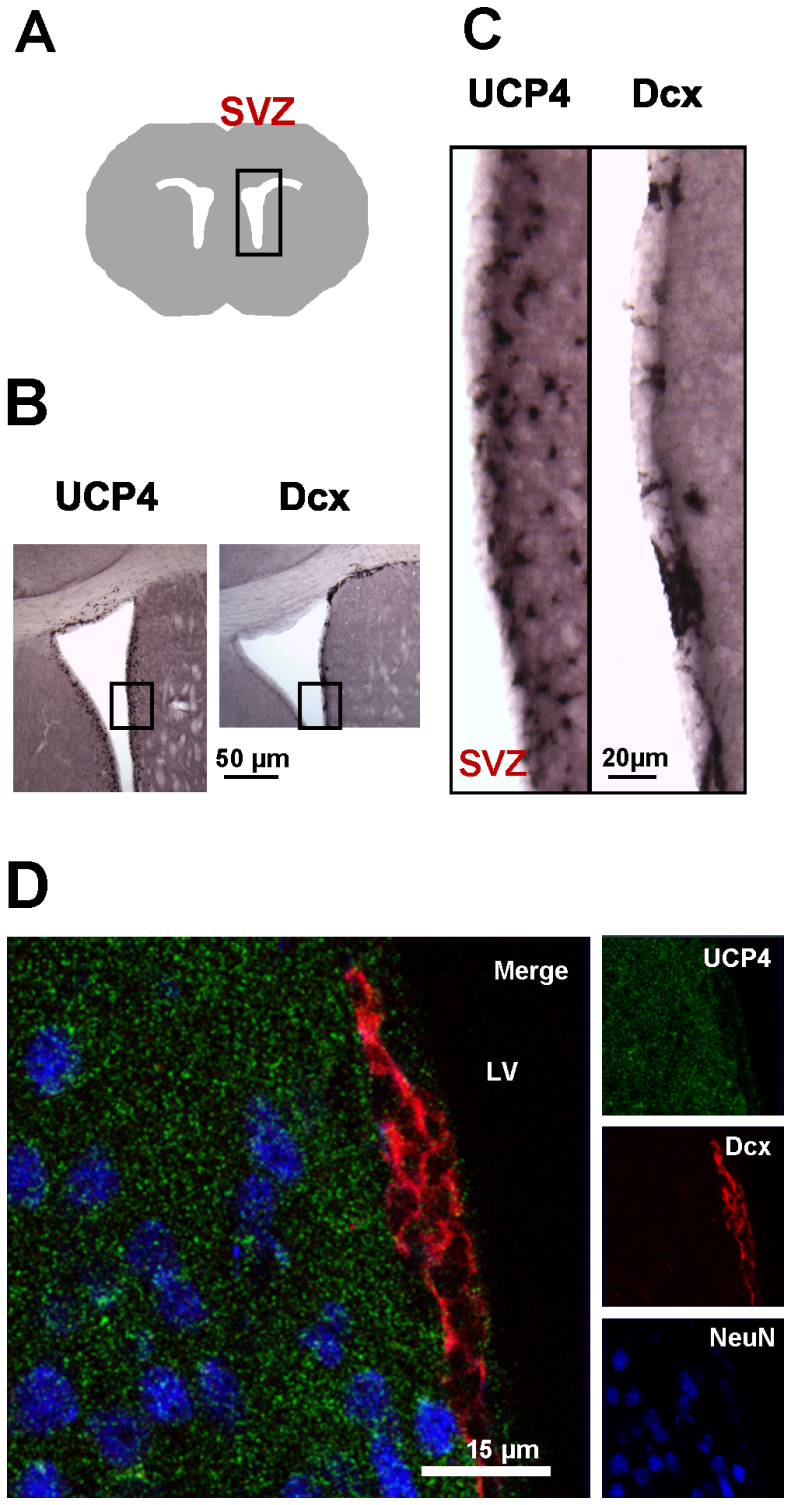
Lack of UCP4 expression in Dcx+/NeuN- neuroblasts in the adult subventricular zone (SVZ). A. Schematic drawing illustrates the localization of the SVZ of the lateral ventricle in adult mouse brain. B–C. Light microscopy analysis of the representative immunohistostained sample shows the distribution of UCP4- and Dcx-positive cells within the SVZ in 50 µm thick coronal sections of adult mouse brain. D. Representative CLSM images of UCP4 (green), Dcx (red) and NeuN (blue) stained with respective antibodies and visualized using Alexa 488, Alexa 594 and Alexa 633 fluorescent dyes.

It is known that adult brain neurogenesis occurs in restricted regions such as in the subventricular zone of the lateral ventricle (SVZ) and the subgranular zone of gyrus dentatus (SGZ) [31], [32]. We analyzed these zones using specific antibodies against UCP4, neuronal migration protein doublecortin (Dcx) and neuronal marker NeuN. We performed an immunohistochemical staining of coronal brain sections from 5 month old C57BL/6 mice (Fig. 5 A, B). The IHC revealed the positive immunoreactivity in SVZ for the antibodies against UCP4 and Dcx (Fig. 5B and C). To identify the cell type responsible for UCP4-immunoreactivity in SVZ, we performed confocal scanning microscopy (Fig. 5D). The co-localization of triple-stained brain sections showed that all mature neurons, which were detected by anti-NeuN antibody (blue), were positive for UCP4 (green). In contrast, no UCP4 expression was observed in Dcx-positive adult stem cells (red).

Unfortunately, there is no appropriate antibody against UCP2. Our antibody is only reliable when used specifically in WB. Therefore, we were not able to test the expression of UCP2 in this region.

### UCP2 but not UCP4 is present in a neuroblastoma cell line

**Figure 6:**
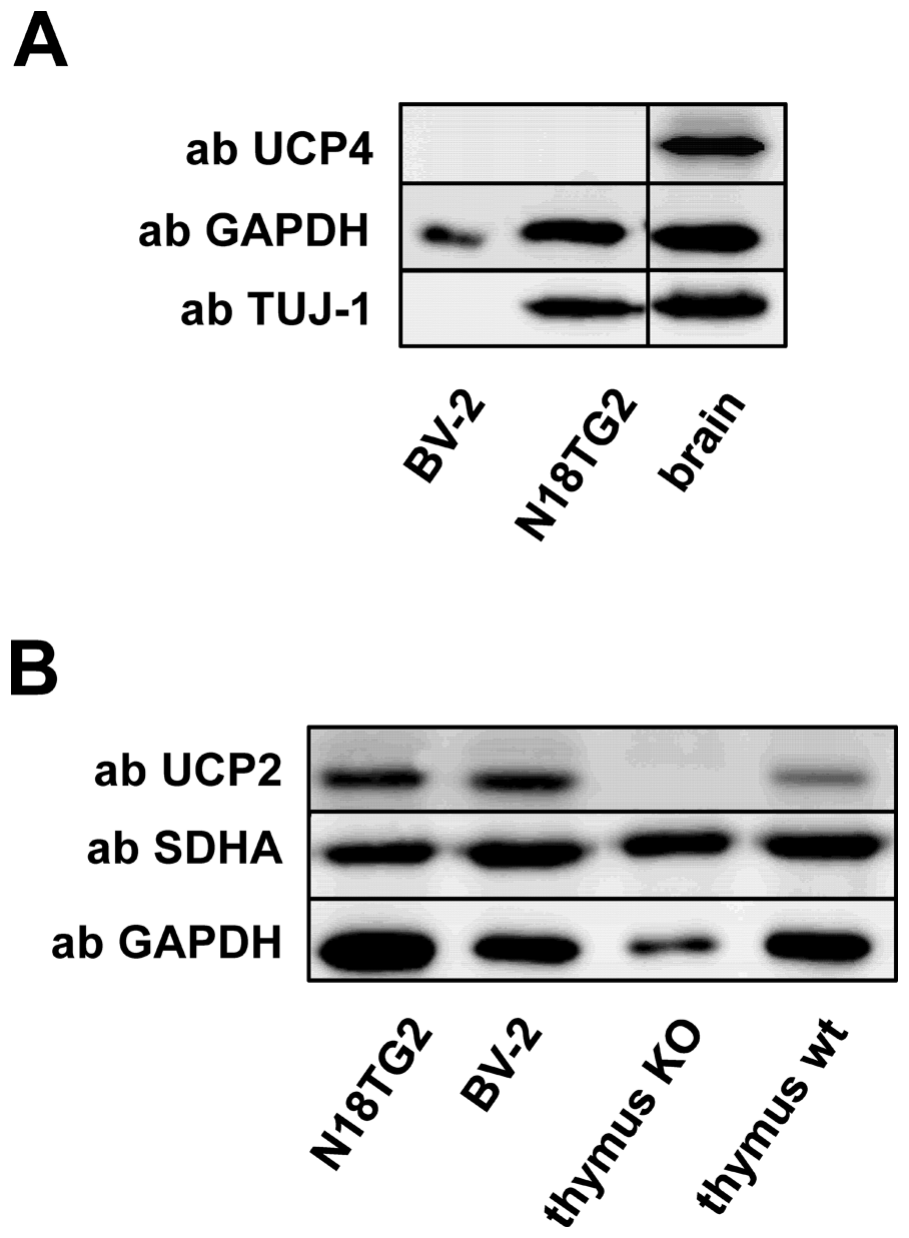
The neuroblastoma cell line N18TG2 expresses UCP2 but not UCP4. A. Representative Western blot analysis of UCP4 expression in the murine neuroblastoma cell line N18TG-2 and murine microglial cell line BV-2. Mouse brain tissue was used as a positive control for the antibody against UCP4. B. Representative Western blot analysis of UCP2 expression in the murine neuroblastoma cell line N18TG-2 and murine microglial cell line BV-2. Thymus of UCP2 knockout (KO) and wild type (wt) mice were used as negative and positive controls for the antibody directed against UCP4. Gels were loaded with 20 µg protein per lane. Cells from at least three different passages were analyzed in each experiment.

The function of neuronal UCPs and other proteins are often analyzed in immortalized cell lines. The data presented in Fig. 6 clearly show that UCP4 is not expressed in the mouse neuroblastoma cell line N18TG2, although these cells were positive for the neuronal marker TUJ-1 (Fig. 6A). We also could not detect UCP4 in the murine microglia cell line BV-2, which as anticipated, was negative for the neuronal marker TUJ-1. In contrast, UCP2 was prominently present in both cell lines (Fig. 6B) which supports the hypothesis that UCP2 is expressed in highly proliferative cells [12], [20], [33]. Although this result was to be expected, to our knowledge, UCP2's presence in neuroblastoma cells was experimentally demonstrated for the first time. UCP1, UCP3 and UCP5 were neither detected in the N18TG2 nor in the BV-2 cell lines.

## Discussion

In this work, we, for the first time, performed the comprehensive analysis of UCP2 and UCP4 expression in mouse embryonic stem cells (mESC) during their differentiation into neural cells. We revealed that only undifferentiated highly proliferative stem cells express UCP2. After the initiation of neuronal differentiation, UCP2 protein levels dropped abruptly and did not appear at later time points of differentiation, whereas UCP2 mRNA remained nearly constant throughout the differentiation period. Moreover, we could not detect UCP2 in murine embryonic brain tissue after the start of neurogenesis.

Results presented in this work clearly show that both proteins (but not mRNA of these proteins) do not occur in the same cell type at the same time (Table.1). Although UCP2 mRNA is ubiquitously distributed among different cell types, detectable amounts of UCP2 at the protein level seem to be present only in fast proliferating cells which are metabolically supported by aerobic glycolysis such as activated lymphocytes, macrophages, hematopoietic stem cells and cancer cells [12], [15], [19], [20], [22], [33]. These cells have in common that they change their metabolisms aiming to increase their proliferative potential and the ability to synthesize new molecules “on demand” [34]–[36].

**Table 1:** Summary of UCP2, UCP4 and UCP5 distribution at mRNA and protein levels, obtained in our laboratory using RT PCR and WB with positive (recombinant proteins) and negative (knockout mouse for UCP2) controls.

	mRNA ratio to GAPDH					
	+++ > 0.1			protein detection		
	0.1 > ++ > 0.005			in 20 µg total protein		
	0.005 > + > 0.001					
	UCP2	UCP4	UCP5	UCP2	UCP4	UCP5
brain	++	++	++	O	X	O
spinal cord	++	++	+	O	X	O
heart	++	+	O	O	O	O
skeletal muscle	+	O	O	O	O	O
BAT	n.a.	n.a.	n.a.	O	O	O
WAT	+++	+	+	O	O	O
spleen	+++	+	+	X	O	O
thymus	+++	++	+	X	O	O
lungs	+++	++	+	(X)	O	O
stomach	+++	+	+	(X)	O	O
intestine	n.a.	n.a.	n.a.	(X)	O	O
liver	++	O	O	O	O	O
kidney	++	+	+	O	O	O
mononuclear immune cells	n.a.	n.a.	n.a.	X	O	O
neurons	O	+	+	O	X	O
astrocytes	++	+	+	(X)	X	O
microglia	+++	O	O	X	O	O
stem cells	+++	++	+	X	O	O
neuroblastoma cells	n.a.	n.a.	n.a.	X	O	O

Crosses and circles indicate the positive and negative tested tissues of adult mice and murine cells, respectively. X indicates tissues in which UCP2 was always detected. (X) indicates tissues in which UCP2 was often but not always detected or tissues where the protein abundance variation was very strong. Non-analyzed tissues are marked as n.a. Data were published in [12], [16], [17] and in the present paper.

Whereas fast proliferating cells use aerobic glycolysis to promote cell growth, neurons, also known to have high metabolic demands and strong dependence on glucose as an energy source, rely on a permanent ATP supply by oxidative phosphorylation [34], [37]–[39]. In support of the above-mentioned differences in cells metabolic backgrounds, the distribution pattern of UCP4 found exclusively in neurons and neurosensory cells [16], [17], [40], [41] seems to be completely opposite to that of UCP2. We detected the highest level of UCP4 expression around day 14 of neuronal differentiation and this coincides with the termination of their differentiation. In our previous work we revealed a similar tendency for UCP4 abundance in mice embryos at different developmental stages [16].

UCP2's presence in other cell types cannot be excluded either, especially in those which are able to acutely induce aerobic glycolysis from a proliferative and metabolic standstill, e.g. intestinal cells and fibroblasts in the course of logarithmic growth [42], [43]. Recently it was shown that stimuli for endothelial cell proliferation evoke strong up-regulation of UCP2 [44]. The direct proof for protein up-regulation is elusive, because of difficulties in isolating the amount of primary cells sufficient for WB analysis. However, this hypothesis would explain the reports of several groups including our own, concerning the trace of UCP2 in lung and intestine [12], [45] as an alternative to the previously given explanation that relies on the invasion of immune cells [12].

The detection of UCP2 in cell lines (neuroblastoma, BV-2) shown in this work may indicate that metabolism of neuroblastoma cells is not comparable to that of native neurons. Therefore, caution is required in studies of cell metabolism when using corresponding cell lines that express UCP2 as a characteristic feature. Moreover, the overexpression of UCP2 in cells that normally lack this protein may present inaccurate results because of its intervention in metabolism, which is not typical for the primary cells.

The role of UCP2 in mitochondria of fast proliferating cells is still uncertain. A recent finding showed that by its artificial expression, UCP2 hindered the differentiation of pluripotent stem cells [20]. Zhang et al. proposed that UCP2 prevents mitochondrial pyruvate oxidation and promotes the metabolism of aerobic glycolysis. Previously, it was suggested that UCP2 facilitates the development of tumors by promoting the metabolism of cancer cells [46], [47]. In contrast, a UCP2 knockout mouse was found to have had a higher potential of cancer development as the wild type [48]. Pecqueur et al. reported that T-cells lacking UCP2 have an increased proliferation rate, but only in the presence of high glucose [33]. UCP2 is clearly essential for the metabolism of highly proliferating cells and therefore necessarily down-regulated with changes in metabolism, as we have shown in the present work. UCP2 function may thereby be a protection of proliferating cells in case of substrate shortages [33].

The importance of UCP2 for proliferating cells may explain the ubiquitous presence of UCP2 mRNA in all tissues, because it ensures the possibility of sudden proliferation as required during development and growth, due to increased activity, uncontrolled proliferation in cancerogenesis and repair after tissue injury. The latter would explain, why UCP2 is often reported to be up-regulated in a range of neurodegenerative and ischemic disease such as multiple sclerosis, seizure, stroke, brain trauma, and ischemia [49]–[56].

### Summary

The presence of UCP2 in the mouse embryonic stem cell clone D3 and neuroblastoma cells evaluated in the present study is in agreement with our formerly proposed distribution pattern of UCP2. Based on the presented and previous results, demonstrating the expression of UCP2 in embryonic cells, cancer cells, (activated) lymphocytes and macrophages, we suggest that the presence of UCP2 may be characteristic for cells with high proliferative and anabolic potential. The expression of UCP2 in adult neurons under physiological conditions seems very unlikely, due to their different metabolic features. Our present results indicate a new pathway for the study of UCP2/UCP4 functions.

## Supporting Information

Figure S1 **Representative Western blot for UCP3 antibody validation.** 25 µg total protein from skeletal muscles (SkM) of UCP3 knockout mice (KO) and their wild type controls (Wt) were loaded per lane. 3 months old mice were tested (n = 3).
(TIFF)
